# Detecting Nano-Scale Vibrations in Rotating Devices by Using Advanced Computational Methods

**DOI:** 10.3390/s100504983

**Published:** 2010-05-18

**Authors:** Raúl M. del Toro, Rodolfo E. Haber, Michael C. Schmittdiel

**Affiliations:** 1 Centro de Automática y Robótica, Consejo Superior de Investigaciones Científicas, Ctra. Campo Real km 0.200, Arganda del Rey 28500, Madrid, Spain; E-Mails: rhaber@iai.csic.es (R.E.H.); mschmit@iai.csic.es (M.C.S.); 2 Escuela Politécnica Superior, Universidad Autónoma de Madrid, C/ Francisco Tomás y Valiente 11 28049, Madrid, Spain

**Keywords:** vibration measurement, signal processing algorithm, frequency domain analysis, nanotechnology

## Abstract

This paper presents a computational method for detecting vibrations related to eccentricity in ultra precision rotation devices used for nano-scale manufacturing. The vibration is indirectly measured via a frequency domain analysis of the signal from a piezoelectric sensor attached to the stationary component of the rotating device. The algorithm searches for particular harmonic sequences associated with the eccentricity of the device rotation axis. The detected sequence is quantified and serves as input to a regression model that estimates the eccentricity. A case study presents the application of the computational algorithm during precision manufacturing processes.

## 1. Introduction

The growth in recent decades of the nanotechnology area has led to the emergence of new challenges for researchers and engineers, due to the need for the development of sensors and devices to characterize physical phenomena or quantify the properties and characteristics of materials at the nano-scale [1]. The achievable accuracy of devices and instruments related to this field requires state of the art technology and ground breaking research. Contributions to the field of precision manufacturing will have a positive impact on sectors such as medicine, industrial, communications, aviation, aerospace and defence, among others.

Electro-mechanical devices that are usually employed in precision manufacturing processes typically have nonlinear behavior for most representative physical variables, low signal-to-noise ratio, strong influence of environmental factors, the high presence of uncertainty and a huge volume of data generated at high frequencies. Therefore, conventional methods often cannot be applied for the characterization of physical phenomena in these devices. However, the use of advanced signal processing strategies, and experimental modelling techniques are useful and feasible ways for studying physical processes at these devices.

Recent researches on precision manufacturing are focused on the development of rotary actuators for positioning with high accuracy [2,3]. The performance of these devices is enhanced by the introduction of control systems to reduce the influence of environmental factors such as temperature [4] and employing magnetic actuators to isolate external vibration [5]. The use of multi-sensory monitoring strategies, such as acoustic emission [6] and vibration sensors [7] is chosen to improve device capabilities. Moreover, due to vibration signals with low signal to noise ratio, much attention has been focussed on the use of advanced computational algorithms for signal processing [8–10].

The main contribution of this paper is the development of a method, based on a computational algorithm for signal analysis in the frequency domain combined with a regression model, to detect nano-scale vibration, and to estimate the eccentricity at the spinning axis of ultra precision rotation devices. This knowledge can be applied to reducing systemic errors, thus reducing manufacturing time.

This paper is organized as follows: Section 2 presents an introduction about the use of ultra precision rotation devices in manufacturing operations and a mathematical model of vibrations in rotation devices; Section 3 describes an experimental analysis to study the relationship between vibrations and shaft eccentricity in an ultra precision rotation device; Section 4 explains the implementation of an algorithm to find sequences of harmonics in the frequency spectrum of a vibration signal and, as an example, introduces a regression model to estimate the eccentricity of the rotation device. Finally, some conclusions about the work results are shown in Section 5.

## 2. Ultra Precision Rotation Devices in Manufacturing Processes

In precision manufacturing processes, the use of rotation devices with ultra precision requirements [11] is mandatory. Operations like milling, turning, drilling, *etc.*, to produce components with micro or nano scale features, are performed by machine tools with nanometric resolution of their positioning axes. The precision in the movements is mainly achieved by employing linear motors and spindles with hydrostatic or magnetic bearings. These techniques avoid the stiction and reduce the influence of vibrations, friction and thermal deviation. Some technical specifications of an air bearing spindle employed for milling, turning and grinding operations are shown in Table 1.

In spite of the state-of-the-art mechanical and computational technology, inadequate dynamic behavior of a positioning system affects the dimensional accuracy of manufactured parts. The appearance of vibrations can cause unwanted motion in any axis. The dynamic forces that arise during the rotation of devices, such as the spindle of an air bearing [12], reflect these unwanted movements. Forces increase due to the dynamic mass imbalance of the spindle, which generates an eccentricity on its rotation axis and corresponding vibrations. These vibrations have a direct influence on the precision of the manufactured part.

### 2.1. Eccentricity and Vibrations due to Mass Imbalance in Rotors

Eccentricity in the shaft of a rotating device occurs when its center of mass differs from its geometric center [13]. One of the most common causes is the device mass imbalance, which is produced, mainly, by unequal distribution of masses of its components. Eccentricity in the shaft can generate dynamic forces that cause vibrations synchronous to the rotation frequency of the device.

**Figure 1.**
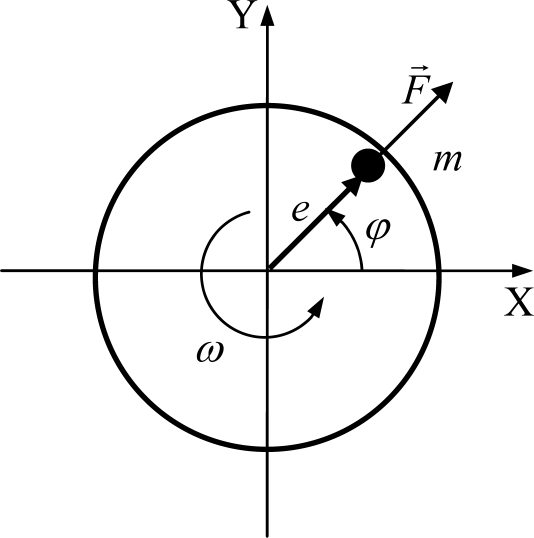
Schematic of a flat rotor and imbalanced mass.

Figure 1 shows a diagram of a flat rotor and a point with mass *m* causing imbalance. The imbalance mass is also characterized by an eccentricity *e* to the rotor axial axis and angle φ. If the rotor has an angular velocity ω, the amplitude of the resultant force *F* and its components, *Fx* and *Fy*, due to the imbalance are calculated as [13]:

(1) $$\begin{array}{c} \mathrm{Fx}=\mathrm{me}\omega^{2}\text{cos}\varphi \\ \mathrm{Fy}=\mathrm{me}\omega^{2}\text{sin}\varphi \end{array}\;\;\Rightarrow \;\;F=\mathrm{me}\omega^{2};\varphi =\omega t$$

**Figure 2.**
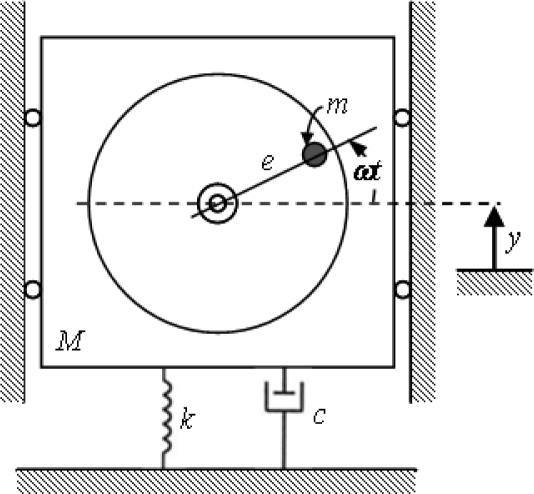
Physical model of a rotary device with an imbalanced mass [14].

These forces constitute a harmonic excitation to the rotating device, causing vibrations in the same direction and frequency of the excitation force [14]. In order to mathematically estimate these vibrations, the rotating device could be considered as a spring-mass-damper system, with coefficient of viscosity *c* and elasticity *k*. For simplicity of analysis, initially the excitation only is considered in one direction (see Figure 2). The elasticity and viscosity of others rotor components (e.g., the bearings) are not considered directly in this analysis.

If *y* is the displacement of the non-rotational mass (*M* − *m*) from the equilibrium position and the displacement *y*_m_ of the unbalanced mass *m* is determined as:

(2) $$y_{m}=y+e\;\text{sin}\;\omega t$$

The general equation of motion is represented by:

(3) $$(M-m)\frac{d^{2}y}{{\mathrm{dt}}^{2}}+m\frac{d^{2}}{{\mathrm{dt}}^{2}}y_{m}=-\mathrm{ky}-c\frac{\mathrm{dy}}{\mathrm{dt}}$$

Equation (3) can be simplified as follows:

(4) $$M\frac{d^{2}y}{{\mathrm{dt}}^{2}}+c\frac{\mathrm{dy}}{\mathrm{dt}}+\mathrm{ky}=(\mathrm{me}\omega^{2})\;\text{sin}\;\omega t$$

The excitation input to the system is the unbalance force component in the *y* direction (*Fy*). The solution of above equation has two parts, the homogeneous and the particular solution. The homogeneous solution describe the transient behavior of the system and it is a free vibration that can be under damped, over damped or critically damped [14]. At steady state, the response of the system is characterized by the particular solution of the equation, which is an oscillatory vibration of the same frequency as the excitation *Fy* with amplitude *Y* and phase φ [14]:

(5) $$y=Y\text{sin}(\omega t-\phi );\;\;\;\begin{array}{c} Y=\frac{\mathrm{me}\omega^{2}}{\sqrt{{\left(k-M\omega^{2}\right)}^{2}+{\left(c\omega \right)}^{2}}}=\frac{\frac{\mathrm{me}}{M}{\bar{\omega }}^{2}}{\sqrt{{\left(1-{\bar{\omega }}^{2}\right)}^{2}+{\left(2\zeta \bar{\omega }\right)}^{2}}}\\ \phi ={\text{tan}}^{-1}\frac{c\omega }{k-M\omega^{2}}={\text{tan}}^{-1}\frac{2\zeta \bar{\omega }}{1-{\bar{\omega }}^{2}} \end{array};\;\;\;\begin{array}{c} \bar{\omega }=\;\frac{\omega }{\omega_{n}};\;\;\omega_{n}{}^{2}=\frac{k}{M}\\ 2\zeta \omega_{n}=\frac{c}{M} \end{array}\;\;$$

where ζ is the damping factor of the system and ω_n_ its natural frequency.

From the second derivative of *y*, the acceleration of motion could be expressed as:

(6) $$\ddot{y}=-\omega^{2}Y\text{sin}(\omega t-\phi )$$

The above equations represent the relationship between the eccentricity, caused by the imbalanced mass, and vibrations that take place in a rotating device. The amplitude of both vibration and its acceleration is proportional to the unbalance mass amount and its eccentricity.

## 3. Experimental Analysis

In order to experimentally study the relationship between vibrations and shaft eccentricity, an experimental platform has been installed on a spindle model SP-150 from Precitech Inc, mounted on an ultra precision lathe. These types of machines are employed for finishing operations in curved and flat surfaces of both brittle and ductile materials, with very low error tolerances. Components (e.g., an optical lens) with arithmetic average surface roughness below 10 nm and few hundred nanometers of form accuracy can be manufactured.

**Figure 3.**
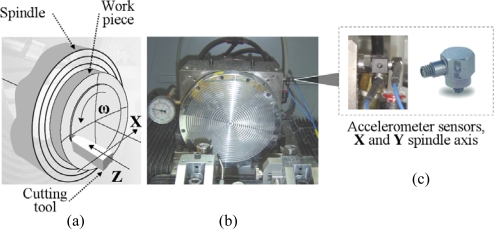
(a) A workpiece attached to the spindle and machine axes. (b) Ultra precision spindle model SP-150. (c) Piezoelectric accelerometer sensors model 352C15.

Vibration signals are measured with two accelerometer sensors rigidly attached to the spindle housing (see Figure 3). The sensor model is 352C15 from PCB Piezotronics, which has a sensitivity of 10 mV/g and a bandwidth of 12 kHz. The vibration signals of X and Y spindle axes are acquired and processed with the high performance processor PXI-8187 from National Instruments, with a sample frequency of 50 kHz.

The ultra precision lathe, model Nanoform 200 from Precitech Inc, is located within an industrial environment and part of a functioning production line, necessitating that the experimental platform does not interfere with the manufacturing process.

The eccentricity reference’s value is obtained from a measurement system embedded into the lathe’s computer numerical control (CNC). Amplitude and phase of this value correspond to the maximum eccentricity position of spindle shaft, which are depicted on the graphical user interface of the CNC. These values can only be obtained prior to each manufacturing operation. For the experimental analysis only the amplitude of eccentricity is used as reference value.

In order to analyze the relationship between vibration level and shaft eccentricity, different operation conditions of the spindle have been considered: not rotating, rotating at different speeds and different eccentricity values. Some of the operating conditions for the experiments are shown in Table 2. The eccentricity has been caused by manually adding imbalanced masses on the spindle.

From expert operator criteria, the acceptable tolerance for spindle shaft eccentricity is 50 nm, and values lower than this number, are insignificant.

**Figure 4.**
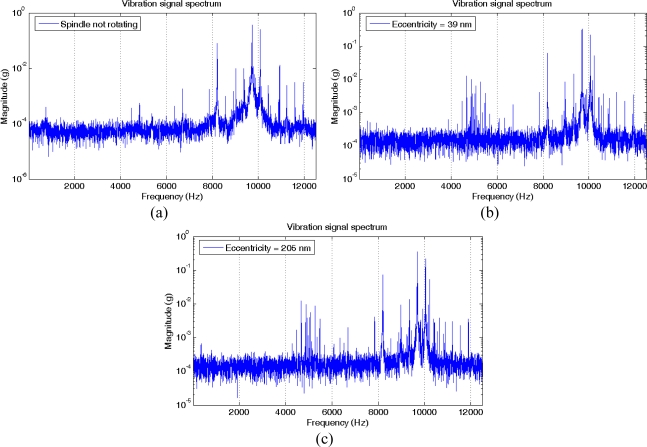
Magnitude spectra of the X-axis vibration signal. (a) Spindle not rotating. (b) Spindle rotating at 1000 r/min, eccentricity of 39 nm. (c) Spindle rotating at 1000 r/min, eccentricity of 205 nm.

Only the X-axis spindle vibration signal is analyzed in this study. The fast Fourier transform (FFT) is applied to this signal, generating a frequency spectrum. A sample size of 50,000 samples is used for each transform, thus the frequency step in the spectrum is equal to 1 Hz. Figure 4 shows the magnitude spectrum in logarithmic scale for three situations: a non-rotating spindle and a spindle rotating at 1000 r/min (16.7 Hz) but with two different values of eccentricity (39 and 205 nm).

**Figure 5.**
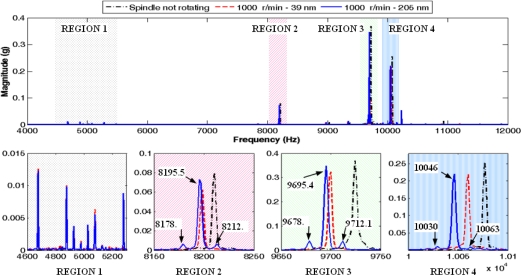
Frequency spectrum expanded around the main harmonic components.

The spectra are quite similar at first glance, except in a region around 5 kHz where there are harmonics related to the rotation. New harmonics also appear near the frequency of the main harmonics. Figure 5 depicts the three analyzed cases and the spectrum expanded in four frequency regions. The first region corresponds to the harmonics close to 5 kHz and the other regions are related to the frequencies around the main harmonics of the spectrum, with the largest harmonic within the third region. As it is shown in regions 2, 3 and 4, there are two new sideband harmonic components for the signal collected with 205 nm in eccentricity that are not present in the other two spectra. In the case of the third region, these new harmonics and the main harmonic have a frequency of 9678.7, 9695.4 and 9712.1 Hz. The difference between them is just 16.7 Hz, which corresponds exactly to the spindle rotation frequency. In the regions 2 and 4, also appear new sideband harmonic components. Moreover, within the first expanded region, this event does not occur.

The above analysis in the frequency domain is the basis for formulating the following hypothesis: if a harmonics sequence separated by the rotation frequency of the device exists in a frequency range around one of the main harmonics of the spectrum, then the device shaft has an eccentricity due to its mass imbalance.

**Figure 6.**
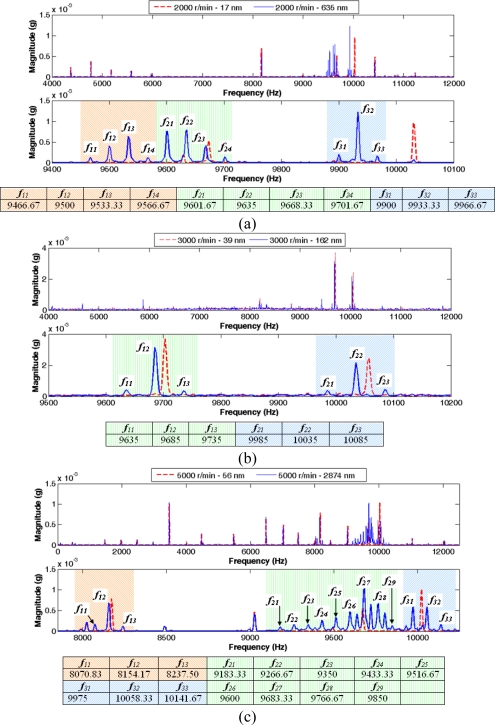
Frequency spectrum for six cases and harmonics sequences.

For a better understanding of the formulated hypothesis, Figure 6 illustrates six more cases of the vibration signal magnitude spectrum, three different rotation frequencies and two values of eccentricities: 33.33 Hz (2,000 r/min), eccentricities of 17 and 635 nm (Figure 6a); 50 Hz (3,000 r/min), 39 and 162 nm (Figure 6b) and a rotation frequency equals to 83.33 Hz (5,000 r/min), eccentricities values of 56 and 2874 nm (Figure 6c). For each rotation frequency, the figure depicts the spectrum at a frequencies range with the more relevant information and the spectrum expanded within a frequencies interval close to the main harmonics, showing in the case of the biggest eccentricity value, the harmonics sequences and the frequency of each component. According the figure, it can be verified that on each harmonics sequence the frequency difference between two consecutive harmonics is equal to the rotation frequency.

## 4. Harmonics Sequences Detection (HSD) Algorithm

**Figure 7.**
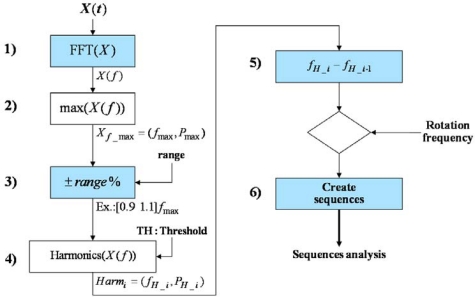
Block diagram of the harmonic sequences detection algorithm.

An algorithm to find sequences in the spectrum of the vibration signal is designed from the previous study and hypothesis. The main parameters of the algorithm are the rotation frequency of the device and the desired frequency search range. Figure 7 shows the block diagram of the developed algorithm.

The main steps of the algorithm depicted in Figure 7 are:

1. The DC component of the measured signal is removed by subtracting the mean value and then transformed into the frequency domain by applying an FFT, ***X*(*f*) = *FFT*(*X*(*t*) − *X̄*)**. Also a Hann or Hamming window can be applied to the measured signal.
2. Find the amplitude of the main harmonic component, ***P***_max_, and its corresponding frequency, ***f***_max_.
3. Set the desired frequency range around ***f***_max_ to perform the search, taking into account the sampling frequency of the signal (***SF***) and avoiding signal aliasing, this range could be defined as:
  (7) $$(1-\mathrm{range})f_{\text{max}}\leq f\leq (1+\mathrm{range})f_{\text{max}};\;\;0<\mathrm{range}<\frac{\mathrm{SF}}{4f_{\text{max}}}-1$$
4. To detect the harmonics within the frequency range, any peak detector function can be applied.
5. Calculate the distance in frequency between the harmonics up to a maximum window given by the spindle rotation frequency.
6. Harmonics separated to the spindle rotation frequency, are counted and grouped into the corresponding sequence.

In order to calculate the Fourier transform, the algorithm uses a number of samples (*N_fft*) from the signal ***X(t)***, proportional to the ratio between the sampling frequency of the signal (*SF*) and the spindle rotation frequency (*RF*):

(8) $$N\_\mathrm{fft}=\frac{\mathrm{NxRPM}\cdot \mathrm{SF}}{\mathrm{RF}}$$

where *NxRPM* defines the resolution of the spectrum and must be an integer. Increasing *NxRPM* can improve the harmonic detection, but also increases the noise when calculating the FFT.

For each harmonic sequence found, the total number (*PA*), the largest magnitude (*BPG*) and the sum of all magnitudes (*HSPG*) are recorded. The number of harmonic components and their amplitudes are then quantified.

Furthermore, on each sequence, the relationship between the main harmonic and the rest is calculated, obtaining a measure of the relative power between the harmonics:

(9) $$\mathrm{PRA}=1-\frac{\mathrm{BPG}+\mathrm{BP}}{\mathrm{HSPG}+\mathrm{BP}}$$

where *PRA* is the power ratio and *BP* is the magnitude of the main harmonic at the spectrum range.

The eccentricity in the spindle shaft can be estimated on the basis of the information obtained from the frequency analysis of the vibration signal. The next subsection proposes a simple model based on regression techniques to estimate the eccentricity in the spindle shaft.

### 4.1. Experimental Regression Model

**Figure 8.**
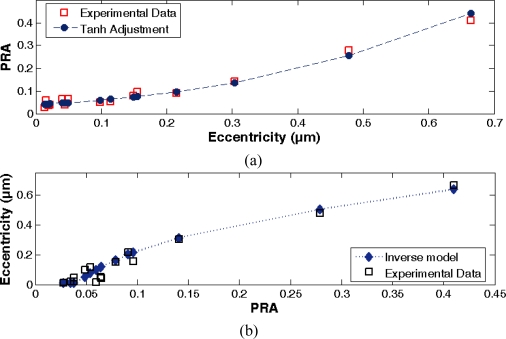
Experimental data and fitted curves, (a) direct model, *PRA versus* Eccentricity, (b) inverse model, Eccentricity *versus PRA*.

A direct model that is represented by a hyperbolic tangent function with a correlation coefficient (R^2^) equal to 0.98 is adjusted from an experimental set of data by applying regression techniques and the HSD algorithm. The model [see Equation (10)] relates the eccentricity (*UL*) in the spindle shaft with the power ratio of the detected sequence around the main harmonic of the spectrum. Inverting the direct function, it is obtained a model that estimates the eccentricity and uses the power ratio as input [see Equation (11)]. Figure 8 shows the power ratio of harmonic sequences and its corresponding eccentricity value from the experimental data and the adjusted models.

In order to adjust both model equations, several values of spindle rotation frequency and its corresponding shaft eccentricity, are taken into account. The experimental dataset is detailed in Table 3, where the second column refers to the power ratio between harmonics of the detected sequence.

(10) $$\mathrm{PRA}=\frac{1}{2}\left[\text{tanh}(m\;\cdot \mathrm{UL}+n)+1\right]$$

(11) $$UL=\left\{ \begin{array}{ll} UL_{min}, & PRA\leq PRA_{min}\\ \frac{1}{m}\left[\frac{1}{2}\text{ln}\left(\frac{PRA}{1-PRA}\right)-n\right], & PRA>PRA_{min} \end{array}\right.;PRA_{min}=\frac{1}{2}\left[\text{tanh}(m\;\cdot UL_{min}+n)+1\right]$$

In the above equations *m* and *n* are linear fit coefficients, adjusted initially to 2.220126 and −1.59696 respectively. Moreover, the equation parameters *UL_min_* and *PR_min_* are defined as minima specifications for the eccentricity and the power ratio, respectively. The minimum value *UL_min_* is set to 0.010 μm according to the range of the experimental dataset. These parameters are introduced to take into account the sensitivity of the piezoelectric sensor and the vibration attenuation because of the spindle mechanical properties. From the physical model of the spindle vibration [see Equation (5)], it can be see that the damping factor ζ can attenuate the amplitude of the vibration due to the eccentricity, then, the sensor will not detect a vibration signal change lowest than its sensitivity.

In order to evaluate the model performance, the absolute error between the model estimation and the measured eccentricity is calculated by the following equation:

(12) $$\mathrm{ERA}=\frac{\left|{\mathrm{UL}}_{E}-{\mathrm{UL}}_{M}\right|}{{\mathrm{UL}}_{M}}\cdot 100\%$$

The last column of Table 3 shows the error for each test, given an average error of 27%, which could be considered an acceptable performance of the fitted model. Indeed, this error is relatively high for the accuracy requirements because the uncertainty of estimation at the lower ranges affects the full range model performance. The main cause of this uncertainty is either, the low magnitude of vibrations due to the rotation device stiffness or the low sensitivity of the piezoelectric sensor employed for vibration measurement. Furthermore, this level of error is considered acceptable from an industrial precision manufacturing standpoint [15].

## 5. Conclusions

In this work a computational method that incorporates a signal processing strategy is proposed to estimate the eccentricity in ultra precision rotation devices, due to its inertial mass imbalance. The eccentricity is estimated from steady state vibrations caused on the device structure, during the rotary movement. These vibrations are measured employing piezoelectric accelerometer sensors. The use of piezoelectric accelerometer sensors instead of displacement sensors, such as capacitive and inductive sensors is justified due to the versatility of this sensor type under several working environment conditions.

The harmonic components related with these vibrations are identified by applying advanced spectral analysis algorithms to the vibration signals. The quantification of the harmonics power, serves to estimate the eccentricity of the rotating device. This procedure enables the design of new control systems in order to compensate for nano-scale vibrations, and thus improving accuracy and precision of rotation devices.

## Figures and Tables

**Table 1.** Technical specifications of spindle model SP-150 from Precitech Inc.

**Maximum Speed**	7000 r/min
**Axial Stiffness**	175 N/μm
**Radial Stiffness**	87 N/μm
**Motion accuracy**	Axial/Radial ≤ 25 nm
**C-axis feedback resolution**	0.13 arc-sec
**C-axis position accuracy**	+/−2 arc-sec

**Table 2.** Operating conditions of the experiments for the study of spindle vibrations.

**Spindle speed [r/min]**	**Eccentricity [nm]**
1000	14, 192, 205, 309
2000	17, 635
3000	17, 39, 98, 162, 1358
5000	56, 2874

**Table 3.** Experimental data, eccentricity estimation and error of regression model.

**Spindle rotation frequency (Hz)**	***PRA***	***UL****_M_***^(1)^(μm)**	***UL****_E_***^(2)^(μm)**	***ERA*(%)**
16.67	0.031	0.013	0.010	23.08
	0.032	0.016	0.010	37.50
	0.013	0.021	0.010	52.38

33.33	0.053	0.049	0.071	45.20
	0.078	0.149	0.162	8.87

50.00	0.039	0.018	0.010	44.44
	0.055	0.077	0.078	1.69
	0.116	0.304	0.263	13.53

66.67	0.013	0.017	0.010	41.18
	0.084	0.155	0.182	17.30
	0.266	0.478	0.491	2.66

83.33	0.021	0.019	0.010	47.37
	0.053	0.114	0.069	39.65
	0.074	0.215	0.152	29.47
	0.446	0.665	0.670	0.78

		**Mean Error**		**27.01**

(1) Measured eccentricity.

(2) Estimated eccentricity.
